# Real-time investigation of a *Burkholderia cenocepacia* bacteraemia outbreak in a Vietnamese intensive care unit

**DOI:** 10.1016/j.jhin.2025.04.003

**Published:** 2025-04-18

**Authors:** A.T.K. Nguyen, V.K. Phuong Linh, D.T. Huong, P.T. Kieu, V.V. Phat, H.T. Tuyen, Q. Nguyen, N.H. Hien, N.T. Diem Trinh, H.N. Hon, N.T. Binh, P.T. Phuong Thao, T. Quang, N.T. Thu Van, P.T. Ngoc Lan, Y. Mo, D.L. Paterson, G. Thwaites, L. Thwaites, P.T. Duy

**Affiliations:** a Oxford University Clinical Research Unit, Ho Chi Minh City, Vietnam; b Hospital for Tropical Diseases, Ho Chi Minh City, Vietnam; c Trung Vuong General Hospital, Ho Chi Minh City, Vietnam; d Centre for Tropical Medicine and Global Health, Nuffield Department of Clinical Medicine, Oxford University, Oxford, UK; e ADVANCE-ID Network, Saw Swee Hock School of Public Health, National University of Singapore, Singapore, Singapore; f Infectious Diseases Translational Research Programme, Yong Loo Lin School of Medicine, National University of Singapore, Singapore, Singapore; g Division of Infectious Diseases, University Medicine Cluster, National University Hospital, Singapore, Singapore; h Mahidol-Oxford Tropical Medicine Research Unit, Faculty of Tropical Medicine, Mahidol University, Bangkok, Thailand

**Keywords:** *Burkholderia cenocepacia*, Nosocomial outbreak, Bacteraemia, Hospital-acquired infection, ICU

## Abstract

**Background:**

The *Burkholderia cepacia* complex (Bcc), a group of environmentally ubiquitous bacteria, are inherently resistant to antiseptics and antibiotics. Bcc can proliferate in pharmaceutical products, resulting in nosocomial outbreaks. However, Bcc is often dismissed as blood culture contaminants, and precise identification of Bcc species remains challenging in resource-limited settings, leading to under-treatment and delay in outbreak detection. This paper reports the first identified Bcc bacteraemia outbreak in a Vietnamese intensive care unit (ICU).

**Methods:**

In June 2023, a Bcc bacteraemia outbreak was acknowledged by the hospital authorities after examination of clinical and microbiological evidence. A comprehensive investigation was performed, encompassing epidemiological and clinical review, environmental sampling, whole-genome sequencing (WGS), and implementation of enhanced infection prevention and control (IPC) measures.

**Results:**

The bacteraemia outbreak involved 19 ICU patients between May and August 2023. The causative bacteria were identified as *B. cenocepacia* belonging to a novel sequence type, and did not carry any acquired antimicrobial resistance genes. Although these organisms were susceptible to the commonly used antibiotics, the outbreak was associated with a high case fatality rate. Three *B. cenocepacia* isolates were also found in used syringes for drug infusion in three cases, exhibiting a genomic link to the outbreak cluster. Enhanced IPC measures targeting aseptic techniques in handling intravenous medications resulted in termination of the outbreak.

**Conclusions:**

WGS plays a crucial role in outbreak control, particularly for under-studied opportunistic pathogens. This work also highlights key gaps in IPC measures, species identification, and treatment of Bcc infections, warranting further research to improve hospital prevention and treatment strategies.

## Introduction

Nosocomial outbreaks occur worldwide but are often under-reported in low- and middle-income countries (LMICs), where they are likely more common due to overcrowded patient populations and insufficient hospital infection control resources [1]. Additional challenges are the lack of robust surveillance of hospital-acquired infections, reliable molecular tools in species identification, and outbreak confirmation of uncommon opportunistic bacterial pathogens. Collectively, hospitals in LMICs remain highly vulnerable to nosocomial outbreaks, often leading to severe outcomes.

The *Burkholderia cepacia* complex (Bcc), a group of Gram-negative, non-fermenting bacilli encompassing more than 22 species [2], are typically found in the natural environment, but they have also been identified as important opportunistic pathogens [3,4]. Bcc bacteria can cause severe infections in critically ill and immunocompromised individuals, and patients with chronic lung diseases [5–7]. Among Bcc species, *Burkholderia cenocepacia* is the most virulent species, accounting for the largest number of Bcc infections worldwide [4,6,7].

Bcc bacteria are inherently resistant to antiseptics, disinfectants and various antibiotics, including carboxypenicillins, first- and second-generation cephalosporins, polymyxins and aminoglycosides. These organisms can survive and proliferate in both sterile and non-sterile medical products, including intravenous drugs and solutions, nasal sprays, mouthwash and skin solutions, making them a major cause of nosocomial out-breaks globally [8]. According to a systematic review, 111 nosocomial outbreaks have been reported across hospitals in Europe, North America and Asia between 1971 and 2019, of which 35.1% occurred exclusively in intensive care units (ICUs) [9]. In the documented Bcc outbreaks, the major infectious syndromes were bacteraemia and pneumonia [7,10,11]. The source of outbreaks was identified in 73.9% of the reported outbreaks, of which 53.2% were linked to contamination of medical solutions, medications and disinfectants [9]. Moreover, Bcc outbreaks may originate from contaminated medical devices, such as extracorporeal membrane oxygenation water heater devices or haemodialysis systems [10,12].

In the clinical setting, Bcc bacteria are often regarded as contaminants in blood cultures for several reasons. Firstly, they are commonly present in the environment, which increases the likelihood of sample contamination during collection, handling or processing. Secondly, Bcc bacteria have low pathogenicity and infrequently cause infections, except in individuals with cystic fibrosis [4]. Furthermore, Bcc bacteria are capable of colonizing the respiratory tract of patients with pre-existing lung conditions [4]. Importantly, the failure of determining Bcc species in the microbiology laboratory can lead to misidentification of true Bcc pathogens rather than contamination.

This article reports a real-time investigation of a bacteraemia outbreak of *B. cenocepacia* in an ICU at a tertiary general hospital in Ho Chi Minh City, Vietnam. A rapid response with microbiological and molecular investigations and hospital infection control measures was performed to confirm and contain the outbreak.

## Methods

### Notification of a cluster of Bcc infections in an ICU

The outbreak occurred in a 30-bed general ICU in a 500-bed tertiary acute care hospital that mainly provides intensive and specialized medical care for critically ill patients transferred from other clinical departments in the hospital. Between 29^th^ May and 1^st^ June 2023, this ICU experienced an unusual surge in positive blood cultures for *Burkholderia* spp. among six symptomatically infectious cases (see Microbiological investigation). The diagnoses of these bacteraemia cases were verified by clinicians and microbiological testing. On 1^st^ June 2023, the *Burkholderia* spp. isolates were sent to the Oxford University Clinical Research Unit (OUCRU) laboratory for confirmation and species identification using a matrix-assisted laser desorption/ionization-time of flight (MALDI-TOF) bio-typer (Microflex LT/SH; Bruker, Billerica, MA, USA). All bacterial isolates were identified as *B. cenocepacia*. These findings were communicated to the hospital promptly, serving as an early warning of a potential outbreak in the ICU.

### Real-time investigation and mitigation measures

While Bcc bacteria are commonly considered as blood culture contaminants, the initial cluster of Bcc cases in the ICU was monitored carefully by the hospital authorities. Infection prevention measures were implemented, including enhanced cleaning, personal hand hygiene practices, and the provision of dedicated nurses and medical equipment. Subsequently, Bcc-positive blood cultures from patients clinically diagnosed with bacteraemia continued to rise within the ICU, prompting suspicions of a true outbreak. Between 3^rd^ and 4^th^ June 2023, two additional cases of Bcc infection were identified in the ICU. On 5^th^ June 2023, the hospital officially acknowledged an outbreak of *B. cenocepacia* following a thorough review of clinical and microbiological evidence.

A comprehensive outbreak investigation was initiated, involving the hospital’s ICU ward, microbiology laboratory, and infection prevention and control (IPC) team, in collaboration with OUCRU’s research team. On 5^th^ June 2023, enhanced IPC measures were enacted to curb the spread of Bcc within the ICU, including rigorous hand hygiene protocols, comprehensive cleaning and disinfection of patient areas and medical equipment, environmental decontamination, and rearrangement of patient beds. All cases of Bcc infection were placed under contact precautions, and patient transfers in and out of the ICU were temporarily halted. Furthermore, refresher training on standard practices, including preparation and administration of intravenous fluids, medications, and nutrients for gastrostomy feeding, was intensified to mitigate the risk of Bcc contamination.

While the hospital implemented rigorous measures to control the outbreak, three further cases of Bcc-positive blood cultures were reported between 5^th^ and 9^th^ June 2023.

### Environmental sampling and outbreak containment

On 9^th^ June 2023, extensive environmental sampling was initiated to trace the source of Bcc infections, focusing on high-risk contamination sources within the ICU, including high-touch surfaces, medical products, water sources and air. In total, 97 samples were collected, including 10 air sedimentation samples, 22 swabs from high-touch surfaces, 44 samples from fluid solutions, and 21 samples from medical devices (details in Table S1, see online supplementary material). Liquid samples were collected in sterile containers to avoid contamination. Upon identification of potential sources by whole-genome sequencing (WGS), enhanced IPC measures were intensified to terminate the outbreak. On 29^th^ June 2023, the last new case of Bcc infection was reported, and no further evidence of Bcc transmission or contamination among patients has been observed in the ICU.

### Epidemiological investigation

Although the first cases were detected in late May 2023, a thorough retrospective review of all inpatients with positive microbiological cultures for *Burkholderia* spp. was performed, dating back to January 2023. This review identified a total of 20 Bcc-positive cases, including 19 cases in the ICU and one case in the nephrology and dialysis department. The case from the nephrology and dialysis department was excluded from further investigation due to the absence of an epidemiological link to the ICU cases. Consequently, there were 19 epidemiologically linked patients involved in the Bcc outbreak within the ICU, including 17 definite cases and two probable cases. A definite case was defined as an ICU patient diagnosed with bacteraemia, confirmed by positive *Burkholderia* spp. blood cultures from two separate body sites or from one body site on different occasions, within ± 3 days of onset of clinical signs of bacteraemia. A probable case was defined as an ICU patient with positive microbiological cultures for *Burkholderia* spp., regardless of sample type, during the outbreak period. Clinical and treatment data were reported for the 17 definite cases.

### Microbiological investigation

Microbiological cultures of clinical samples were initially performed in the microbiology department, following routine practices in accordance with Decision 1539/QÐ-BYT of Vietnam’s Ministry of Health on clinical microbiology technical practice guidelines [13].

### Blood culture

Two 8–10-mL blood samples were collected in bottles at different peripheral intravenous sites and inoculated for aerobic blood culture purposes. These bottles were subsequently incubated at 35 ± 2 °C using either the Becton Dickinson BACTEC 9050 or Becton Dickinson BACTEC FX40 automated analyser for up to 5 days. Upon detection of a positive signal, sub-culturing was performed on sheep blood agar (BA) and MacConkey agar (MCA). Organisms isolated from infected patients were identified using API 20E (bioMérieux, Marcy l’Etoile, France), and their antibiotic susceptibility was determined using the disk diffusion method (Kirby–Bauer, Cleveland, OH, USA).

### Tracheal aspirate and catheter tip culture

Cultures of tracheal aspirate samples and catheterized specimens were conducted at the hospital according to routine practices, similar to blood culture. Tracheal aspirate samples were cultured directly on standard agar plates, including BA, MCA and chocolate agar. For catheter tips, the collected fluids were cultured on BA and MCA plates. If the catheter fluids were insufficient for direct culturing, the catheter was immersed in brain heart infusion broth (BHIB) and incubated for 16–20 h to increase the bacterial load before culturing the enriched broth on MCA and BA plates.

### Environmental sample culture

Air samples were collected by sedimentation and incubated at 35 °C ± 2 °C before culturing on BA, MCA and Sabouraud agar plates for colony counting and morphological identification at 24 and 48 h. Other samples (i.e. swabs from high-touch surfaces, antiseptic solutions) were cultured directly on BA, MCA and chocolate agar plates. Samples requiring enrichment were pre-incubated in BHIB before plating on the agar plates. Water samples and medical solutions requiring filtering were subjected to filtration using 0.22-μm vacuum filters (Corning, Corning, NY, USA), followed by rinsing the membrane with phosphate buffered saline and plating on culture agar plates.

### Antimicrobial susceptibility testing

Antimicrobial susceptibility testing (AST) for all clinical Bcc isolates was conducted in the hospital’s laboratory as per routine practice, using the traditional disk diffusion method. AST results were interpreted in accordance with the hospital’s guidelines.

The Bcc isolates were later sent to OUCRU’s laboratory for subculture and species identification using MALDI-TOF. AST of these isolates was re-assessed following the Clinical and Laboratory Standards Institute (CLSI) M100-2023 guidelines. Quality control was performed using *Escherichia coli* strain ATCC 25922. AST was performed with recommended antibiotics, including disks for meropenem, co-trimoxazole and ceftazidime (Oxoid, Basingstoke, USA) and M.I.C.E. strips for levofloxacin (Oxoid).

For any discrepancies in AST results between the hospital’s laboratory and OUCRU’s laboratory, the final results were based on the finding from OUCRU’s laboratory.

### Whole-genome sequencing

WGS was performed on all available Bcc isolates for outbreak confirmation using an Illumina Hiseq platform. In total, 23 *B. cenocepacia* isolates were subjected to WGS, including 20 clinical isolates (16 from blood culture and four from tracheal aspirate culture) and three environmental isolates (three used syringes).

FastQC v0.11.5 was used to perform quality control on all raw Illumina fastq files. SRST2 v.0.2.0 was used to detect acquired antimicrobial resistance (AMR) genes and multi-locus sequence typing (MLST) using the ARG-ANNOT AMR database and the Burkholderia MLST database downloaded from the PubMLST website (https://pubmlst.org/mlst/), respectively [12]. Raw Illumina reads were mapped against the reference genome of *B. cenocepacia* strain 842 (accession number: GCA_001606115.1) using RedDog pipeline v1.10b (https://github.com/katholt/RedDog). Briefly, the RedDog pipeline used Bowtie2 v2.2.3 to map all raw reads, and SAMtools v1.1.3.1 was used to extract high-quality single nucleotide polymorphisms (SNPs), selecting only those that achieved a Phred quality score threshold ≥30 [13]. Core genes present in >95% of isolates were extracted, and 57,497 core SNPs identified in these regions were used to infer the phylogeny. IQ-TREE v.2.0 was used to reconstruct the phylogeny with bestfit TVM+ASC+G4 model, with support from 1000 bootstrap replicates [14]. The pairwise SNP distance matrix for all isolates was calculated using Snp-dists v0.8.2 (https://github.com/tseemann/snp-dists).

### Ethical approval

This outbreak investigation was conducted under the auspices of the hospital authorities. All patient data were anonymized and kept confidential during the investigation. The hospital’s institutional review board waived the requirement for ethical review for this investigation.

## Results

### Epidemiological curve of the B. cenocepacia outbreak

**Figure 1:**
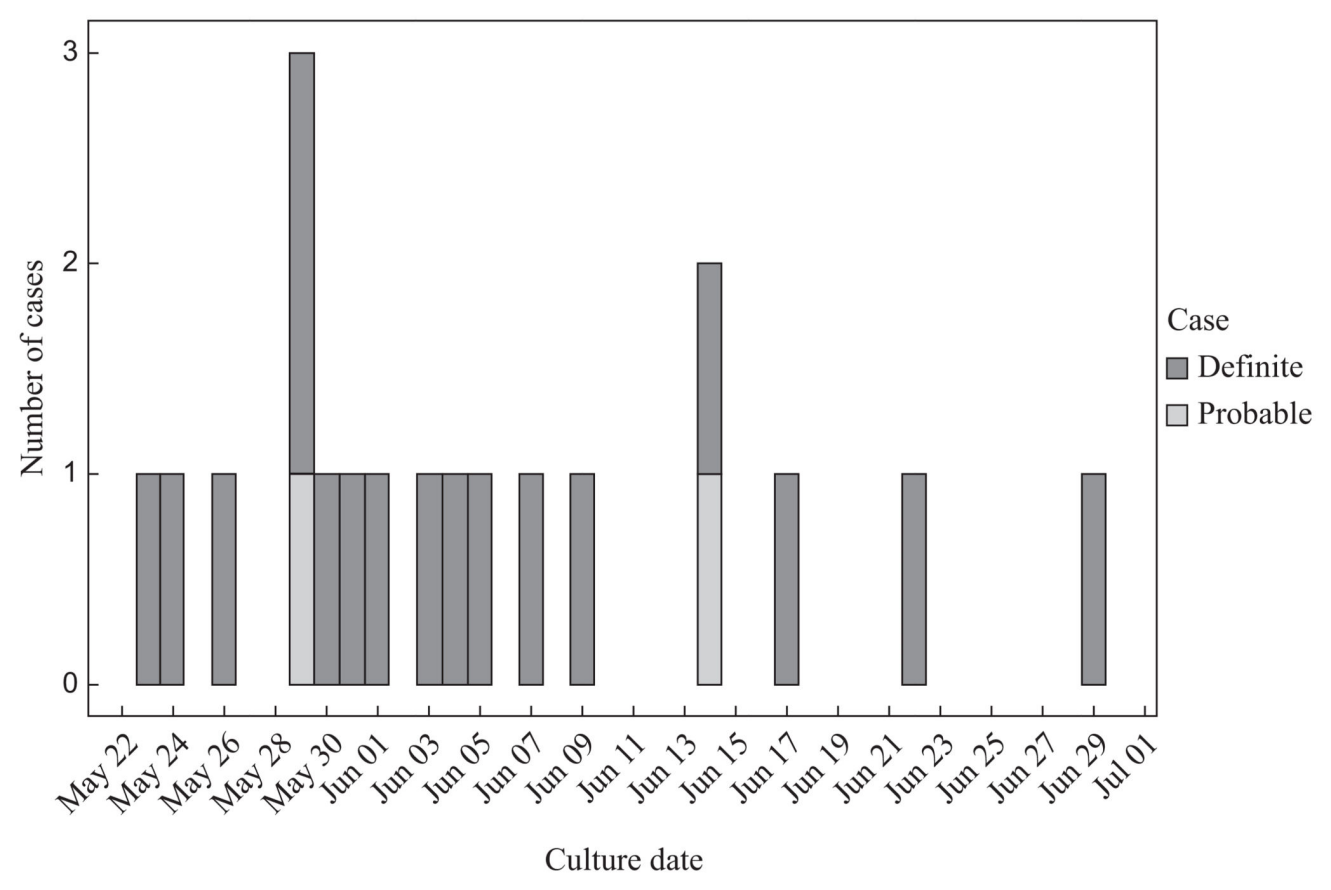
Epidemiological curve of *Burkholderia cenocepacia* outbreak cases in the intensive care unit between May and July 2023.

The epidemiological curve shows the total number of cases related to the Bcc outbreak in the ICU between May and July 2023 (Figure 1). There were 19 outbreak-related patients, with the first case detected on 23^rd^ May 2023 and the last positive case reported on 29^th^ June 2023. The majority of Bcc-infected patients were detected between late May and early June. In total, 17 definite cases were confirmed by positive blood cultures for *B. cenocepacia*, and two probable cases were detected with positive tracheal aspirate and catheter samples.

### Clinical characteristics of definite cases

The baseline characteristics of the 17 *B. cenocepacia* definite cases are described in Table I. The median age of these patients was 68 years (range 48–100 years), and 70.6% were male. Over 80% of patients (14/17) were admitted to the hospital under emergency conditions due to pulmonary disease (52.9%), neurological disease (11.8%), infectious disease (11.8%) and cardiovascular disease (11.8%). The most common comorbidities were hypertension (64.7%), diabetes (29.4%), congestive heart failure (23.5%), ischaemic heart disease (17.6%) and renal disease (11.8%). Fifteen of the 17 Bcc patients (88.2%) were culture-positive for additional organisms, other than Bcc, before or during the outbreak period. The median time from ICU admission to positive blood culture for *B. cenocepacia* was 16 days (range 1–57 days). Fourteen of 17 patients were diagnosed with bacteraemia alone and three patients acquired both bacteraemia and pneumonia. Medical devices were commonly used, including central venous catheters (100%), mechanical ventilators (94.1%), nasogastric tubes (94.1%) and urinary catheters (82.4%). Nearly 95% of positive *B. cenocepacia* cases were given intravenous medication via a syringe pump.

Most patients exhibited clinical signs of bacteraemia (Table II). Fever was recorded in over half of the definite cases. Abnormal white blood cells (e.g. leukocytosis and leukopenia) were identified in 47.1% of patients with *B. cenocepacia*. In all cases, the Sequential Organ Failure Assessment (SOFA) score, C-reactive protein and procalcitonin were elevated. The median antibiotic treatment time used for patients during their hospital stay was 36 days (range 7–103 days).

Various antibiotic groups were prescribed to treat *B. cenocepacia* infections, with a total of 84 prescriptions for both empirical and definitive therapy (Table III). The most common empirical antibiotic regimens were meropenem, amikacin, cefoperazone/sulbactam and vancomycin. Upon the availability of AST results, there was increased use of levofloxacin, cefoperazone/sulbactam, trimethoprim/sulfame-thoxazole and meropenem to manage *B. cenocepacia* infections.

Only three (17.6%) patients survived; 11 cases were discharged for hospice care, two left hospital against medical advice, and one case remained in hospital under ongoing treatment. In terms of day 28 status, 11 patients (64.7%) had died, three patients were alive but required continuous life support, and three patients were lost to follow-up.

### Environmental sampling results

Through the environmental investigation, three *B. cenocepacia* isolates were identified from three separate used syringes in the automatic pump among three definite cases. In these cases, the syringe pumps were used to deliver intravenous fluids, including vasopressors, insulin, NaCl 0.9% and glucose 5%. Following the identification of *B. cenocepacia* in the used syringes, the ICU implemented stricter control on the preparation and delivery of intravenous solutions. Subsequently, no positive cultures with *B. cenocepacia* were detected.

Various opportunistic bacteria were also found in the environmental samples, such as *Acinetobacter baumannii, Klebsiella pneumoniae, Pseudomonas aeruginosa, E. coli, Enterobacter* spp. and *Stenotrophomonas* spp. (details in Table S2, see online supplementary material). However, there was no growth of micro-organisms in the samples of antiseptics, hand sanitizer, ultrasound gel and unopened medical consumables.

### Antimicrobial susceptibility profiles of B. cenocepacia isolates

The antimicrobial susceptibility profiles of all 16 blood isolates and one tracheal aspirate isolate from definite cases were identical to those identified in the used syringes; all of them were susceptible to levofloxacin, meropenem, trimethoprim/sulfamethoxazole and ceftazidime (Table IV). In contrast, the three additional *B. cenocepacia* tracheal aspirate isolates from two definite cases (OB-08 and OB-09) and one probable case (OB-18) exhibited resistance to ceftazidime and meropenem, and intermediate resistance to trimethoprim/sulfamethoxazole.

### Whole-genome sequencing results and outbreak confirmation

**Figure 2:**
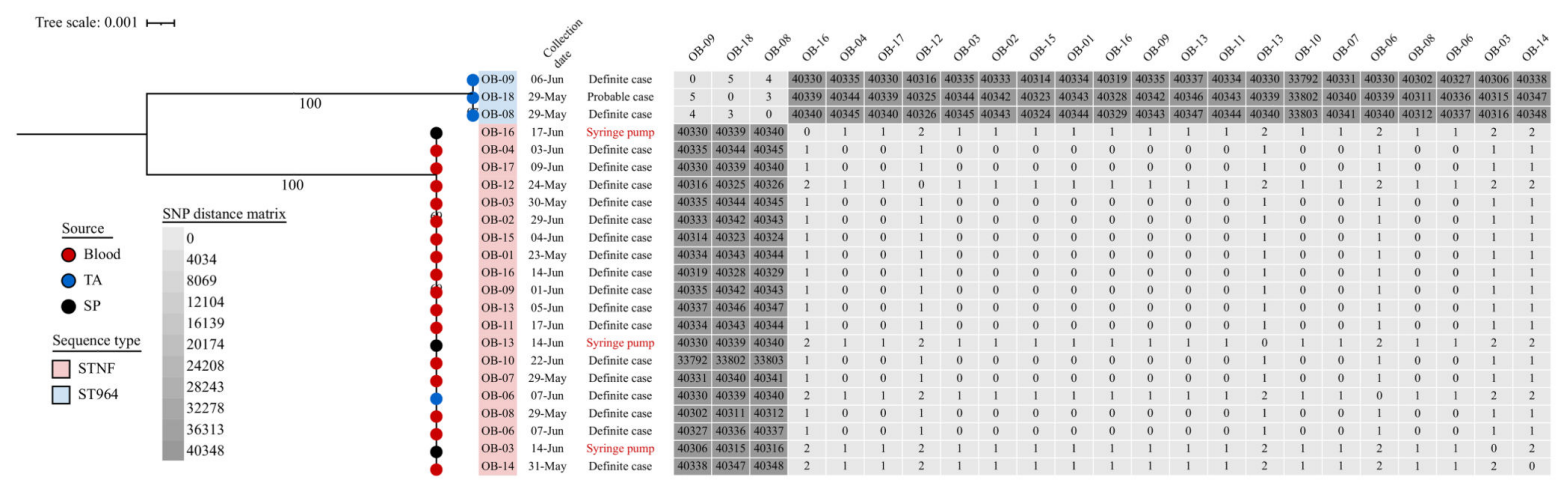
Phylogenetic structure of *Burkholderia cenocepacia* isolates from outbreak cases and used syringes. The phylogenetic tree is mid-point rooted. The types of positive samples are represented by the coloured circles at the terminal nodes of the tree: red for isolates from positive blood cultures, blue for isolates from positive tracheal aspirate (TA) cultures, and black for isolates from positive cultures of used syringes (SP). The outbreak cases from whom samples were collected for microbiological culture are shown at the tip labels. The first column depicts sample collection date; the second column indicates whether the samples were collected from definite cases, probable cases or syringes. The tree scale bar represents the number of single nucleotide polymorphisms (SNPs) per site. The numbers on internal branches show bootstrap support values, with larger numbers indicating higher bootstrap values. The phylogenetic tree is mapped against the pairwise SNP differences between the isolates.

Genomic analysis confirmed that all *B. cenocepacia* isolates identified from blood and used syringes of definite cases belonged to the same sequence type (ST) (Figure 2). This ST showed three allelic differences from its nearest neighbour, ST32, and had not been assigned a number; therefore, it was designated as STNF (NF, not found in MLST database).

Conversely, the three *B. cenocepacia* isolates from tracheal aspirate cultures of two definite cases and one probable case were identified as ST964. These ST964 isolates carried an identical array of acquired AMR genes including *aphA6, bla*_NDM-1_, *bla*_PME-1_, *sul1* and *tetG*, predicted to confer resistance to carbapenems, sulfonamide and tetracycline. No acquired AMR genes were found among the STNF outbreak isolates.

The phylogenetic structure was consistent with the results from genomic analyses, showing that all STNF isolates formed a separate cluster which was distantly related to the ST964 cluster. Furthermore, the STNF isolates identified from definite outbreak cases and used syringes exhibited very limited pairwise SNP distances, ranging from zero to a maximum of two SNP differences. Given that *B. cenocepacia* accumulates mutations at a rate of approximately two SNPs per year [14], these findings revealed that all three environmental isolates obtained from contaminated syringes were genetically linked to the definite outbreak cluster. In contrast, the mean pairwise SNP difference between ST964 and STNF was 40,006 SNPs, highlighting their substantial genetic distance. The ST964 cluster originated from three outbreak cases who were also diagnosed with ventilator-associated pneumonia, and may have resulted from environmental contamination. Following the genomic findings, aseptic handling and administration of intravenous medications were intensified, resulting in termination of the outbreak.

## Discussion

This investigation underscores the importance of hospital surveillance, prompt IPC and the role of WGS in hospital outbreak investigations. This is particularly relevant for complex bacteria such as *Burkholderia* spp., in which traditional detection and typing methods often have low sensitivity. WGS has revolutionized bacterial disease surveillance and outbreak responses; however, in LMIC settings such as Vietnam, its adoption in the clinical setting is very limited. There is a pressing need for training and capacity building on genomics and its utilities in the clinical context, including clinical diagnostics and IPC programmes. Without strong collaboration and coordination between the IPC team, clinical microbiologists and molecular biologists, such Bcc outbreaks may be overlooked.

This investigation not only confirmed and contained the *B. cenocepacia* bacteraemia outbreak, but also revealed major technical and knowledge gaps in the detection and treatment of these infections. Bcc infections in healthcare settings are largely understudied/under-reported in many LMIC settings. This stems from the challenge of distinguishing clinical infection from colonization/contamination; for example, in this outbreak, *B. cenocepacia* bacteraemia had been regarded as blood culture contamination for 2 weeks before being acknowledged as true infection. Some patients were co-infected with other pathogens or may have been receiving treatment for other infections, which could have led to a delay in recognizing Bcc as the true pathogen. Furthermore, there is a lack of diagnostics at the species level, which has major implications in clinical management. Species identification is critical because Bcc comprises >22 species, exhibiting differing epidemiological and pathological features, and varying susceptibility to antibiotics both *in vitro* and *in vivo* [2]. For example, among Bcc species, only a few are commonly detected in human infections, such as *B. cenocepacia, B. multivorans, B. stabilis* and *B. vietnamensis* [15]. Species misidentification may lead to inappropriate antimicrobial therapy, increased antimicrobial resistance, and in-vivo emergence of more virulent variants [16].

Furthermore, although the *B. cenocepacia* outbreak isolates were susceptible to the commonly used drugs at the hospital, including trimethoprim-sulfamethoxazole, meropenem, levofloxacin and ceftazidime, the final clinical outcome was poor. One possible reason for this is the weak correlation between invitro antimicrobial susceptibility results and the clinical response of patients [17]. Currently, there is no consensus or guideline on effective antimicrobial therapy for Bcc infections, highlighted by the fact that while CLSI has released cut-off values for in-vitro antibiograms, the European Committee on Antimicrobial Susceptibility Testing has not [18]. Bcc bacteria are known to adapt rapidly to antimicrobial drugs during the course of treatment, leading to potential failure of treatment for outbreak cases despite adequate antibiotic selection following AST [17]. Additionally, the poor outcome of outbreak cases is possibly due to the comorbidities and severity of medical conditions of ICU patients, who had high SOFA scores, prolonged hospitalization, prolonged ICU stay and increased duration of mechanical ventilation. Another factor is that Bcc patients can acquire co-infections with multiple bacterial pathogens during the same infection episode, or may be exposed to other bacterial pathogens either before or after the Bcc infection, further complicating treatment responses and outcomes. To address the gaps in diagnostics and treatment of Bcc infections, there is a need for further large-scale genomic and clinical surveillance of Bcc infections to gain awareness and clinical consensus of Bcc nosocomial infections, as well as to improve species identification and determine the level of correlation between in-vitro and in-vivo results.

Throughout the outbreak investigation, the possible routes of bacterial transmission between outbreak cases were investigated, including shared staff and shared invasive medical equipment and materials, but no evidence of direct or indirect transmission was found. In contrast, three *B. cenocepacia* isolates were identified from three separate used syringes in automatic syringe pumps, which were genetically linked to the outbreak cluster. Automatic syringe pumps are commonly used to deliver a precise amount of fluid, such as medication or nutrients, to a patient through a catheter or intravenous line. Previous studies have shown that syringe pumps can be contaminated by bacteria through various mechanisms, including improper handling of syringes, inadequate disinfection, or fluid backflow into the pump [19,20]. Syringes that are reused or not sterilized properly may introduce bacteria into the pump or multi-dose intravenous fluids, potentially leading to infections/outbreaks [21–23]. This research highlights the importance of following aseptic practices and procedures in handling and administration of intravenous medications to avoid bacterial contamination and prevent infections. Furthermore, healthcare staff should be aware of the risks associated with contaminated medical products and devices, whether extrinsic or intrinsic, and should receive training on preventive measures to avoid future outbreaks. This study also found a number of opportunistic hospital-acquired infection pathogens from high-risk contamination sources in the ICU, such as wash basins, bed controllers, bed handles, medical trays and trolleys, air sedimentation, door handles, ventilators, syringe pumps, and gastric feeding tubes. These findings have led to improvement in infection control procedures across the ICU and other hospital departments.

In conclusion, this article reports a bacteraemia outbreak caused by *B. cenocepacia* involving ICU patients in a tertiary hospital in Vietnam. Although the *B. cenocepacia* isolates displayed in-vitro susceptibility to common treatment drugs, the outbreak was associated with a high case fatality rate. The source of the outbreak was not identified, but the infection prevention measures on the preparation and administration of intravenous medications were likely compromised. This work highlights the need to raise awareness among healthcare staff about the potential sources of Bcc contamination, and true nosocomial infections caused by these organisms. Further research is warranted to improve detection methods and species identification, and generate robust clinical evidence on the effectiveness of antimicrobial therapy.

## Supplementary Material

Table S1, Table S2 Supplementary data to this article can be found online at https://doi.org/10.1016/j.jhin.2025.04.003.

## Figures and Tables

**Table I:** Baseline characteristics of all definite cases of *Burkholderia cenocepacia* infection

Characteristic	Patients (*N* = 17)
Age, median (range), years	68 (48–100)
Male sex	12 (70.6%)
Transfer from another hospital	10 (58.8%)
Admission type	
Emergency	14 (82.4%)
Elective	3 (17.6%)
Reason for admission	
Pulmonary	9 (52.9%)
Neurological	2 (11.8%)
Infectious disease	2 (11.8%)
Cardiovascular	2 (11.8%)
Gastrointestinal	1 (5.9%)
Endocrine/metabolic	1 (5.9%)
Comorbid conditions^a^	
Hypertension	11 (64.7%)
Congestive heart failure	4 (23.5%)
Ischaemic heart disease	3 (17.6%)
Diabetes	5 (29.4%)
Renal disease	2 (11.8%)
Chronic pulmonary disease	2 (11.8%)
Cancer/leukaemia	2 (11.8%)
Time to positive *B. cenocepacia* culture, median (range), days	
From hospital admission	21 (6–57)
From ICU admission	16 (1–57)
Positive *B. cenocepacia* culture sample	
Blood	17 (100%)
Tracheal aspirate	3 (17.6%)
Infectious syndrome related to *B. cenocepacia*	
Bacteraemia alone	14 (82.4%)
Bacteraemia and pneumonia	3 (17.6%)
Devices used before *B. cenocepacia* culture^a^	
Central venous catheter	17 (100%)
Mechanical ventilation	16 (94.1%)
Urinary catheter	14 (82.4%)
Gastric feeding tube	16 (94.1%)
Syringe pump	16 (94.1%)
Time to discharge from positive	11 (5–22)
*B. cenocepacia* culture, median (range), days	
Discharge status	
Hospice care	11 (64.7%)
Alive	3 (17.6%)
Left against medical advice	2 (11.8%)
Ongoing^b^	1 (5.9%)
Day 28 status	
Died	11 (64.7%)
Alive – not back to normal activities	3 (17.6%)
Unknown	3 (17.6%)

ICU, intensive care unit.

a Individual patient can have more than one underlying chronic disease and more than one medical device at the same time.

b The patient remained hospitalized.

**Table II:** Clinical features of bacteraemia caused by *Burkholderia cenocepacia* among definite cases

Characteristic	Patients (*N* = 17)
Fever (>38 °C)	9 (52.9%)
White blood cell count >12,000 or <4000	
Yes	8 (47.1%)
No	9 (52.9%)
SOFA score at infection onset, mean (SD)	8.2 (4.6)
CRP (ng/mL), mean	77.8
Procalcitonin (ng/mL), mean	14.3
Length of hospitalization, median (range), days	31.5 (11–67)
Length of ICU stay, median (range), days	28 (11–67)
Length of mechanical ventilation, median (range), days	31 (0–67)
Duration of antibiotic usage, median (range), days^a^	36 (7–103)

CRP, C-reactive protein; ICU, intensive care unit; SOFA, Sequential Organ Failure Assessment; SD, standard deviation.

a Total duration of antibiotics was calculated, not specifically to the index episodes of bacteraemia or pneumonia caused by *B. cenocepacia*.

**Table III:** Antibiotic therapy for *Burkholderia cenocepacia* bacteraemia among definite cases

Number of prescriptions for used antibiotics	Patients (*N* = 17)	
	Empirical antibiotics^a^	Definitive antibiotics^b^
Total	21	63
β-lactam/β-lactamase inhibitor		
Piperacillin/tazobactam	2	4
Cefoperazone/sulbactam	3	10
Cephalosporins		
Fourth generation		
Cefepime	-	1
Cefpirome	-	1
Third generation		
Cefoperazone	1	-
Ceftazidime	-	1
Carbapenems		
Meropenem	4	10
Imipenem	-	2
Fluoroquinolones		
Levofloxacin	2	9
Ciprofloxacin	1	5
Aminoglycosides		
Amikacin	4	5
Sulfonamides		
Trimethoprim/sulfamethoxazole	1	5
Glycopeptides		
Vancomycin	3	5
Polymyxins		
Colistin	-	4
Oxazolidinones		
Linezolid	-	1

a Antibiotics started ≤24 h before infection onset, and received for ≥48 h from the time of infection onset.

b Antibiotics used for ≥48 h from day of antimicrobial susceptibility testing report.

**Table IV:** Antimicrobial susceptibility profile of all available *Burkholderia cenocepacia* isolates

Outbreak ID	Case	Sample	CAZ-CLSI 2023	SXT-CLSI 2023	MEM-CLSI 2023	LEV-CLSI 2023
OB-01	Definite	Blood	S	S	S	S
OB-12	Definite	Blood	S	S	S	S
OB-07	Definite	Blood	S	S	S	S
OB-08	Definite	Blood	S	S	S	S
OB-03	Definite	Blood	S	S	S	S
OB-14	Definite	Blood	S	S	S	S
OB-09	Definite	Blood	S	S	S	S
OB-04	Definite	Blood	S	S	S	S
OB-15	Definite	Blood	S	S	S	S
OB-13	Definite	Blood	S	S	S	S
OB-06	Definite	Blood	S	S	S	S
OB-17	Definite	Blood	S	S	S	S
OB-16	Definite	Blood	S	S	S	S
OB-11	Definite	Blood	S	S	S	S
OB-10	Definite	Blood	S	S	S	S
OB-02	Definite	Blood	S	S	S	S
OB-13	Definite	Syringe pump	S	S	S	S
OB-03	Definite	Syringe pump	S	S	S	S
OB-16	Definite	Syringe pump	S	S	S	S
OB-06	Definite	TA	S	S	S	S
OB-08	Definite	TA	R	I	R	S
OB-09	Definite	TA	R	I	R	S
OB-18	Probable	TA	R	I	R	S

CAZ, ceftazidime; I, intermediate; LEV, levofloxacin; MEM, meropenem; R, resistant; S, susceptible; SXT, trimethoprim/sulfame-thoxazole; TA, tracheal aspirate.

## Data Availability

All raw reads have been deposited in the ENA database (accession number: PRJEB77781).
