# Supplementary material for: Real-time investigation of a Burkholderia cenocepacia bacteraemia outbreak in a Vietnamese intensive care unit
Source: J Hosp Infect. Author manuscript; Available in PMC 2026 Jan 30. (PMC7618689; doi:10.1016/j.jhin.2025.04.003)
Supplement: Table S1, Table S2 — Supplementary data to this article can be found online at https://doi.org/10.1016/j.jhin.2025.04.003. [file EMS212192-supplement-Table_S1__Table_S2.docx]

**Table S1: Environment samples to investigate the *B. cenocepacia* outbreak in the ICU.**

| **Sample types** | | **Number of samples** | **Positions** | **Note** |
| --- | --- | --- | --- | --- |
| **Air (N=10)** |  | 10 | ICU room |  |
| **High-touch surface swabs (N=22)** | | | | |
|  | Doorknob | 1 | Main door |  |
|  | Bed controller | 2 | Bed 10, 19 |  |
|  | Bed handle | 2 | Bed 10, 19 |  |
|  | Medical tray | 1 | Bed 19 |  |
|  | Syringe pump | 2 | Bed 10, 19 |  |
|  | Monitor | 2 | Bed 10, 19 |  |
|  | Medical trolley | 2 | Bed 10, 19 |  |
|  | Ventilator | 2 | Bed 10, 19 |  |
|  | Wash-hand basin | 3 | ICU_Medicines storage area |  |
|  | Surgical wash basin | 5 | ICU |  |
| **Liquid (N=44)** | | | | |
|  | Water | 3 | Tap water from the basin |  |
|  | Drink water | 2 | Bed 10, 19 |  |
|  | Cool and warm (50-60^o^C) water | 2 | Milk preparation room |  |
|  | Using milk | 9 | Bed 4, 5 (x2), 12, 14, 15, 16, 17, 22 |  |
|  | Nutrient milk | 1 |  | Unopen |
|  | Ultrasound gel | 1 |  | Open |
|  | Medical fluids (antiseptics, shower gel, hand sanitizers) | 12 | Bed 10, 19 (open) | Open and unopen |
|  | IV solutions | 14 | Bed 3 (open) | Open and unopen |
| **Medical device (N=21)** | | | | |
|  | Cotton ball | 2 | Bed 27 (open) | Open and unopen |
|  | Cotton ball in blood collection kit | 1 |  | Unopen |
|  | Gloves in blood collection kit | 1 |  | Unopen |
|  | Hetis non-woven pad | 1 |  | Unopen |
|  | Plaid non-woven pad | 1 |  | Unopen |
|  | Sterile gauze pad | 1 |  | Unopen |
|  | Medical dressing | 1 | Bed 14 |  |
|  | Needles | 1 |  | Unopen |
|  | Syringe pump 5cc | 1 |  | Unopen |
|  | Syringe pump 10cc | 1 |  | Unopen |
|  | 3-way stopcock | 1 |  | Unopen |
|  | Wet tissue | 1 |  | Unopen |
|  | Used milk bottle | 1 |  |  |
|  | Gastric feeding tube | 2 | Bed 10, 19 |  |
|  | Syringe pump 50cc for feeding | 2 | Bed 10, 19 |  |
|  | IV fluid in syringe pump | 3 | Bed 10, 17, 19 |  |

**Table S2: List of bacteria from environment samples in the *B. cenocepacia* outbreak in ICU.**

| **Samples** | **Number of samples with presence of bacteria** | **Organism** | **Source of important bacteria** |
| --- | --- | --- | --- |
| Air | 10/10 | *Acinetobacter pavus** | Air in the ICU room |
|  |  | *Agrococcus tereus* |  |
|  |  | *Baccilus cereus* |  |
|  |  | *Baccilus subtilus* |  |
|  |  | *Corynebacterium afermentans* |  |
|  |  | *Cupriavidus respiraculi* |  |
|  |  | *Deinococcus ficus* |  |
|  |  | *Kocuria marina* |  |
|  |  | *Enterococcus faecalis** | Air in the ICU room |
|  |  | *Escherichia coli** | Air in the ICU room |
|  |  | *Lactobaccillus salivarius* |  |
|  |  | *Exiguobacterium aurantiacum* |  |
|  |  | *Micrococcus endophyticus* |  |
|  |  | *Micrococcus flavus* |  |
|  |  | *Micrococcus luteus* |  |
|  |  | *Moraxella osloensis* |  |
|  |  | *Paracoccus yeei* |  |
|  |  | *Psedoclavibacter faecalis* |  |
|  |  | *Pseudomonas aeruginosa** | Air in the ICU room |
|  |  | *Roseomonas mucosa* |  |
|  |  | *Rothia terrae* |  |
|  |  | *Spingomonas faeni* |  |
|  |  | *Staphylococcus arlettae* |  |
|  |  | *Staphylococcus capitis* |  |
|  |  | *Staphylococcus caprae* |  |
|  |  | *Staphylococcus epidermidis** | Air in the ICU room |
|  |  | *Staphylococcus haemolitycus** | Air in the ICU room |
|  |  | *Staphylococcus hominis* |  |
|  |  | *Staphylococcus warneri* |  |
|  |  | *Stenotrophomonas acidaminiphila* |  |
| High-touch surface swabs | 18 /22 | *Achromobacter spp.** | Surgical wash basin |
|  |  | *Acinetobacter baumannii** | Surgical wash basin, bed controller, bed handle, medical trolley |
|  |  | *Acinetobacter indicus** | Surgical wash basin |
|  |  | *Acinetobacter nosocomialis** | Hand wash basin, medical trolley |
|  |  | *Acinetobacter radioresistens** | Medical trolley |
|  |  | *Acinetobacter ursingii** | Wash-hand basin |
|  |  | *Bacillus cereus* |  |
|  |  | *Candida auris** | Medical tray |
|  |  | *Corynebacterium afermentans* |  |
|  |  | *Cupriavidus pauculus* |  |
|  |  | *Delftia acidovorans* |  |
|  |  | *Enterobacter cloacae** | Ventilator, syringe pump |
|  |  | *Enterobacter kobei** | Wash-hand basin |
|  |  | *Enterococcus faecalis** | Bed handle |
|  |  | *Klebsiella pneumoniae** | Wash-hand basin, bed handle, monitor |
|  |  | *Mixta calida* |  |
|  |  | *Moraxella osloensis* |  |
|  |  | *Pantoea septica** | Doorknob |
|  |  | *Pseudomonas aeruginosa** | Wash-hand basin, surgical wash basin |
|  |  | *Pseudomonas kilonensis** | Surgical wash basin |
|  |  | *Pseudomonas stutzeri** | Medical tray |
|  |  | *Roseomonas mucosa* |  |
|  |  | *Rummeliibaccillus stabekissii* |  |
|  |  | *Staphylococcus epidermidis* |  |
|  |  | *Staphylococcus haemolitycus* |  |
|  |  | *Staphylococcus hominis* |  |
| Liquid | 11/14 | *Cupriavidus pauculus* |  |
|  |  | *Brevundimonas aurantiaca* |  |
|  |  | *Pseudomonas aeruginosa** | Surgical wash basin |
|  |  | *Brevibacillus agri* |  |
|  |  | *Pseudomonas stutzeri* |  |
|  |  | *Paenibaccillus naphthalenovorans* |  |
|  |  | *Enterobacter bugandensis** | Using milk |
|  |  | *Kocuria kristinae* |  |
|  |  | *Lactobacillus fermentum* |  |
|  |  | *Lactobacillus gasseri* |  |
|  |  | *Lactobacillus plantarum* |  |
|  |  | *Lactococcus lactis* |  |
|  |  | *Moraxella osloensis* |  |
| Medical devices | 9/21 | *Acinetobacter baumannii** | Gastric feeding tube |
|  |  | *Acinetobacter nosocomialis** | Syringe pump 50cc for feeding |
|  |  | *Acinetobacter soli** | Gastric feeding tube |
|  |  | *Burkholderia cenocepacia** | IV fluid in syringe pump |
|  |  | *Enterobacter asburiae** | Gastric feeding tube |
|  |  | *Escherichia coli** | Gastric feeding tube |
|  |  | *Klebsiella aerogenes** | Syringe pump 50cc for feeding |
|  |  | *Klebsiella pneumoniae* * | Medical dressing, gastric feeding tube |
|  |  | *Kocuria varians* |  |
|  |  | *Lactobacillus fermentum* |  |
|  |  | *Moraxella osloensis* |  |
|  |  | *Proteus mirabilis** | Gastric feeding tube |
|  |  | *Pseudomonas aeruginosa** | Gastric feeding tube, syringe pump 50cc for feeding |
|  |  | *Sphingomonas sanguinis* |  |
|  |  | *Stenotrophomonas maltophilia** | Gastric feeding tube, syringe pump 50cc for feeding |

*considered as important bacteria
